# Are Fe-Based Stenting Materials Biocompatible? A Critical Review of *In Vitro* and *In Vivo* Studies

**DOI:** 10.3390/jfb11010002

**Published:** 2019-12-21

**Authors:** Eleonora Scarcello, Dominique Lison

**Affiliations:** Louvain centre for Toxicology and Applied Pharmacology (LTAP), Université catholique de Louvain, 1200 Brussels, Belgium; dominique.lison@uclouvain.be

**Keywords:** iron-based material, biocompatibility, stent, corrosion, toxicity, ROIs

## Abstract

Fe-based materials have increasingly been considered for the development of biodegradable cardiovascular stents. A wide range of *in vitro* and *in vivo* studies should be done to fully evaluate their biocompatibility. In this review, we summarized and analyzed the findings and the methodologies used to assess the biocompatibility of Fe materials. The majority of investigators drew conclusions about *in vitro* Fe toxicity based on indirect contact results. The setup applied in these tests seems to overlook the possible effects of Fe corrosion and does not allow for understanding of the complexity of released chemical forms and their possible impact on tissue. It is in particular important to ensure that test setups or interpretations of *in vitro* results do not hide some important mechanisms, leading to inappropriate subsequent *in vivo* experiments. On the other hand, the sample size of existing *in vivo* implantations is often limited, and effects such as local toxicity or endothelial function are not deeply scrutinized. The main advantages and limitations of *in vitro* design strategies applied in the development of Fe-based alloys and the correlation with in vivo studies are discussed. It is evident from this literature review that we are not yet ready to define an Fe-based material as safe or biocompatible.

## 1. Introduction

Biodegradable materials are being currently explored as an alternative to permanent implants, in particular for cardiovascular applications such as coronary stents. Indeed, the stent remaining in the artery for the rest of the patient’s life serves no purpose [1]. The scaffold should give strength and support to the artery during the healing process, against the cyclic loading of the blood flow, only for a period between 6 and 12 months after implantation. After this time, the mechanical support is not needed anymore, and, furthermore, the physical permanence of the material could lead to adverse effects such as in-stent restenosis or late thrombosis requiring prolonged antiplatelet therapy [2]. Biodegradable materials were thus designed and developed to support the artery recoil for the needed duration. An ideal biodegradable material for a coronary stent should demonstrate an optimized compromise between degradation and mechanical performances [3]. Polymers from lactic acid, glycolic acid or caprolactone families were first proposed as biodegradable biomaterials. Even if these materials showed excellent biocompatibility as well as ideal degradation rate, their mechanical properties are rather poor and they are unable to fully expand with the use of balloon dilatation [4]. More recently, metals were proposed as biodegradable materials. The idea is to use a metal with a strength close to permanent materials, such as the stainless steels (e.g., 316 L) or Co–Cr alloys. The two main metals that have been investigated are magnesium and iron, selected for their good resistance to traction [5]. The main concern that currently motivates research is to obtain an ideal corrosion rate. *In vitro* degradation assays of Mg-implants in animal models showed an excessive corrosion rate of this metal in vascular stenting applications [3]. Therefore, iron-based materials are considered as an option for novel biodegradable coronary artery stents in view of their lower corrosion rate, and appropriate ductility and strength.

In 2001, Fe-based materials were implanted for the first time in rabbit aortas [6]. Since then, investigators have continued to test Fe-based materials by analyzing corrosion behavior and tissue-material interactions. Many efforts have been made to predict the *in vivo* corrosion rate in acellular tests, from static corrosion assays to complex dynamic systems closer to physiological conditions [7,8,9]. In these pseudo-physiological systems, it is possible to obtain a predetermined shear stress on the sample’s surface close to *in vivo* values, making correlations between acellular and animal models reasonably strong [4].

However, it is fundamental that corrosion does not induce carcinogenic or mutagenic effects [8]. Assessing the biocompatibility is thus a mandatory issue [10]. Biocompatibility is defined not only by low toxicity, inflammation or allergenic potential, but also by no harmful release or retention of materials [3]. Thus, biological evaluations should be systematically conducted *in vitro* and *in vivo*, and safety and effectiveness should be validated by animal experiments with a sufficient sample size.

The aim of this review is to critically review the usefulness and limitations of the most common *in vitro* and *in vivo* test methodologies applied for Fe alloys. Relevant literature and critical appraisal of methods to focus on material biocompatibility were synthesized. Ultimately, this work could give a reference for future investigations with the planning of *in vitro* biocompatibility experiments for the development of Fe-based alloys and subsequent *in vivo* analyses.

## 2. Iron Corrosion and Toxicity

Iron is fundamental for life. The human body contains approx. 45–55 mg Fe/kg of body weight in adult women and men, respectively [11]. Up to 70% of Fe is incorporated in hemoglobin in circulating erythrocytes, almost 30% is stored in hepatocytes and in reticuloendothelial macrophages, and the remaining is in cytochromes, iron-containing enzymes and myoglobin [12]. To compensate for iron losses by sloughed mucosal cells, desquamation, menstruation and other blood loss, the recommended dietary iron intake is 1 to 2 mg per day. Concerning Fe metabolism, this element is transported within the body between the site of absorption and utilization by the plasma glycoprotein transferrin, which reversely binds Fe^3+^ in a turnover of about 30 mg/24 h [13]. About 80% of this Fe is then transported to the bone marrow for hemoglobin synthesis in developing erythroid cells. The macrophages of the reticuloendothelial system phagocytose senescent erythrocytes at the end of their life, and the heme moiety is split from hemoglobin and catabolized enzymatically via heme oxygenase 1 (*HO-1*). Iron is released from the protoporphyrin ring of *HO-1* and returned to the circulation [14]. The remaining 5 mg of the daily plasma iron turnover is principally exchanged with the liver [15].

Iron is critical for vital biochemical activities, e.g., oxygen transport. Redox or hydrolysis reactions regulate most of the biological functions of iron. Despite its fundamental functions for survival, the redox chemistry of Fe underlines paradoxical hazards, mostly based on Fenton chemistry. Fe exists in two principal oxidation states, ferrous (Fe^2+^) and ferric (Fe^3+^), but in aerobic conditions molecular oxygen promotes the shift from ferrous to the more stable ferric state [16]. However, the reduction of dioxygen by Fe^2+^ can generate superoxide anion (O_2_•^−^), which leads to hydrogen peroxide (H_2_O_2_) formation:

Fe(0) → Fe^2+^ + 2e^−^ (1)

Fe^2+^ + O_2_ → Fe^3+^ + O_2_•^−^ (2)

(3) $${\mathrm{Fe}}^{2+}\;+\;O_{2}{•}^{-}\;\overset{2H^{+}}{\to }\;{\mathrm{Fe}}^{3+}\;+\;H_{2}O_{2}$$

H_2_O_2_ ultimately reacts with ferrous iron to produce hydroxyl radicals (HO•) through the well-known Fenton reaction [17]:

Fe^2+^ + H_2_O_2_ → Fe^3+^ + HO• + OH^−^ (4)

HO• is the most powerful oxidant encountered in biological systems, able to induce lipid peroxidation, modification of DNA and oxidative stress. Ferrous ions produced by corrosion of Fe(0) could also generate organic radicals such as peroxyl radicals (ROO•) [11]:

Fe^2+^ + ROOH → Fe^3+^ + HO• + RO• (5)

Fe^3+^ + ROOH → Fe^2+^ + H^+^ + ROO• (6)

The reactive oxygen intermediates (ROIs), i.e., hydroxyl radicals (HO•), superoxide (O_2_•^−^) and hydrogen peroxide (H_2_O_2_), are inevitably produced by aerobic respiration. However, a Fe-based material implanted in an artery corrodes in the same way as described in reactions 1–4 and iron could thus generate a significant amount of ROIs, which will immediately attack and damage cellular macromolecules, and promote cell death and tissue injury [18].

The role of iron in several diseases is well established. Iron overload is associated with colorectal and liver cancer [19]. Inhaled iron compounds have been considered hazardous for the lung due to the fast oxidation of ferrous ions [20]. However, the link between radicals generated from Fe-based material corrosion and the impact on vascular tissue remains almost unexplored in the field of biomaterials. This is rather surprising because the mechanism of metal toxicity related to ROS-production is well-documented in the field of inhaled metal particles [21] and has been explored recently for titanium implants [22]. Tsaryk *et al.* [23] demonstrated that ROS formed by electrochemical processes during Ti implant corrosion induce oxidative stress in endothelial cells.

In the case of Fe-based materials, an hydroxide layer (Fe(OH)_2_) is present on the metal surface immediately after implantation. This layer is permeable to oxygen (contrary to stainless steel materials), allowing the continuity of corrosion during the whole life of the implant [24]. Thus, the implant surface incessantly forms HO•, possibly resulting into prolonged inflammation and unsuccessful healing of the surrounding tissues. In addition, the oxidative stress environment generated by HO• could also worsen damage to and dysfunction of atheromatous tissue [25]. Taking all these oxidative mechanisms into account thus appears crucial for developing new Fe-based implants.

## 3. Assessing the Biocompatibiliy of Fe-Based Materials

The term “biocompatibility” and its definition have been discussed for more than 50 years. Williams in 2014 stated that there is no such thing as a biocompatible material as there is no material with ubiquitous biocompatibility characteristics [26]. Since a material could affect different biological systems in different ways, depending on the context, there is not a uniquely biocompatible material. Thus, biocompatibility refers to a given system, as it is related to the biological tissue within which the material is placed. The degree of success of an implant is determined by reactions at the interface between an implant and the local and systemic environment of the body [27].

The biocompatibility of a medical device is assessed by scrutinizing local and systemic effects on cells, tissue or the whole body through *in vitro* and *in vivo* tests [28]. The preclinical assessment of the biocompatibility of a medical device is defined by a series of standards provided by the International Organization for Standardization (ISO) [29].

### 3.1. In Vitro Experiments

*In vitro* testing is generally defined by the ISO 10993-5:2009 guidelines, which include positive and negative controls, extraction conditions, choice of cell lines and test procedures [29]. Biocompatibility is evaluated by assessing cytotoxicity, haemocompatibility, mutagenesis/carcinogenesis, and cell biofunction [30]. Concerning cytotoxicity, tetrazolium-based assays such as MTT and XTT are the most widely used tools. In these tests, the tetrazolium salt is converted by mitochondrial dehydrogenases to a colored compound, formazan, in a quantity proportional to the number of living cells.

**Figure 1:**
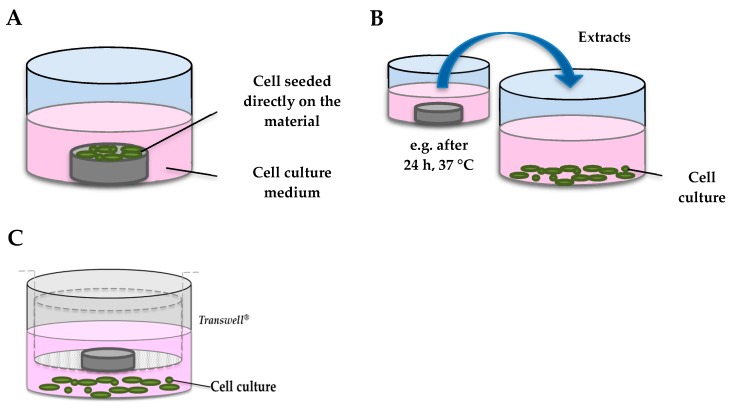
Schematic representation of *in vitro* cytotoxicity tests. (**A**) direct-contact test; (**B**) indirect contact assay; (**C**) exposure to soluble components through a Transwell^®^ insert.

In the direct-contact test, the material should be sterile and in direct contact with cells as indicated in Figure 1A. However, this procedure presents several limitations. First, it is not always clear whether it provides information on cytotoxicity, cell proliferation or attachment. As cells grow on a bulk material, it is more difficult to visually control cells or to characterize a dose-response relationship. Finally, it is harder to assess cells after exposure, e.g., for gene expression. In the indirect contact test (Figure 1B), the material is firstly immersed in an extraction vehicle such as culture medium or physiological saline solution under specific conditions of temperature and timing (normally 24 h/37 °C to mimic physiological conditions). The solution is subsequently centrifuged and cells are exposed to the supernatant, or directly to the bulk corrosion products. This second assay allows isolating the effects of degradation products, but it does not take into account the implication of all the species released by the material during corrosion, especially those with a short half-life, such as ROIs. A third method involves the use of a porous membrane (e.g., Transwell^®^ inserts, Corning, St Louis, MO, USA), with pore sizes ranging from 0.2 to 1 µm, between material and cells (Figure 1C). In this test, only soluble components could reach the cells below. The chemical species released in situ at the surface of the material, such as ROIs, are not taken into account in this setup.

Both procedures intend to represent different situations regarding the impact of an implant on a vascular tissue. The first one is a model of the response of endothelial and smooth muscle cells to the material itself. Indeed, immediately after stenting, there is an initial wound-healing phase where the stent is in contact with both ECs and SMCs. Then, during the largest part of its lifetime, it will be surrounded mainly by SMCs until the formation of a neointima and finally complete and functional endothelium recovery. It thus appears evident that the material should neither induce cytotoxicity or stop the proliferation or migration of cells [31]. Moreover, cells should maintain normal morphology and grow healthily. In order to examine if there is a delay in endothelial cell wound closure, some authors perform the 2D *in vitro* scratch test, which appears particularly relevant because failure of the denuded arterial surface to re-endothelialize leads to increased accumulation of VSMCs and later restenosis [32,33]. To the best of our knowledge, the wound healing assay has never been performed with Fe-based materials.

Below we review the *in vitro* studies available in the open literature on biodegradable Fe or Fe-based materials using massive samples, for cardiovascular applications, tested directly, indirectly or through a porous membrane on cell culture (Figure 1). Studies that investigated the impact of soluble iron salts exclusively, or which did not aim to assess biocompatibility, were not included [34].

A summary of these *in vitro* studies is reported in Table 1 listed by chronological order. Approaches used to investigate the biocompatibility present significant limitations as detailed below.

- The majority of cell viability studies were performed only by exposure to extracts after centrifugation. This simple methodology presents some advantages, e.g., homogeneous concentrations of ion exposure, whereas replicates of insoluble leachates are more problematic to reproduce. However, it assumes that released soluble ions are the main species that can cause cytotoxicity. Few authors compared the consequences of the different procedures on cell cultures. Lin *et al*. [35] compared the three methods for *in vitro* cytotoxicity evaluation, i.e., testing of extracts, direct contact, and the indirect contact method, and they reported completely opposite results. They showed a high fibroblast cytotoxicity after the direct exposure to corrosion particles precipitating during extraction or incubation processes, whereas the exposure to extracts (only iron ions) did not induce cytotoxicity. These results suggest that the supernatant and degradation products in the extracts should be assessed separately in order to identify the exact species responsible for toxic effects. Fagali *et al*. [36] wondered if soluble and insoluble Fe degradation products have different biological impacts, and they concluded that cell toxicity is mainly associated with the presence of insoluble products. The corrosion of Fe-containing materials in a biological environment involves both soluble and insoluble Fe species, stressing the importance of distinguishing the impact of all the components individually. Moreover, as described above, ROIs such as hydroxyl radicals are released during corrosion, which could react with surrounding cells. Due to their short half-life, their possible cytotoxic activity is completely missed in the indirect contact test. We demonstrated in our previous work that only the direct contact between the Fe and cells, and not degradation products, caused cytotoxicity and oxidative stress through HO• release, as confirmed by the protective role of catalase [37].
- The surface of bulk materials is often pretreated prior to cellular testing. Grinding processes differ from 1000 to 4000 mesh papers and are only seldom followed by diamond paste polishing. Surface treatments such as mechanical polishing or electrolytic polishing enhance the corrosion resistance, while an increased surface roughness amplitude with a low surface organization increases the corrosion rate [38]. It has been previously demonstrated that surface roughness amplitude has an influence on the corrosion rate of biodegradable materials and consequently the concentration of released species [39,40]. Moreover, Martin *et al*. [41] demonstrated that the surface roughness of Ti implants alters osteoblast proliferation, differentiation, and matrix production *in vitro*. Therefore, to simulate the biodegradation process of implants and the related released species, the surface treatment of test samples should be as close as possible to clinical products [42].
  Cells used for cytotoxicity assays are not always relevant for endovascular implants (e.g., BALB/3T3 fibroblasts). The stent is mainly in contact with endothelial and smooth muscle cells during its lifetime, simultaneously during the initial wound-healing phase, or exclusively with SMCs after neointima formation. Selecting a given cell type implies a specific scenario for the remodeling of the artery as each cell type has a different role and sensitivity. Some authors examined the response of cell types separately and found preferential cellular sensitivity [35,43,44,45]. As restenosis is one of the principal adverse effects of stent implantation, some authors argue that elective cytotoxicity to VSMCs could antagonize restenosis by reducing excessive vascular cell proliferation [34]. However, this probably represents oversimplification because neointimal proliferation is a complex process involving the interaction with different cell types, including endothelial cells, platelets and macrophages. Additionally, it should be demonstrated that VSMC cytotoxicity is not associated with other damage such as oxidative stress, or consequent dysfunction.
  Few authors have used human cell lines, highly relevant for the development of human endovascular implants, or primary cell lines, instead of animal cells, which are easier to handle and give more consistent results but are less closely related to the clinical situation [7,18,35,45]. Finally, so far, Fe-based materials have not been tested in co-culture or 3-dimensional models, which might better predict the *in vivo* response in humans.
- Cell viability is often assessed by a single assay. Interference and disturbances in viability assays are, however, likely to happen as previously reported for materials other than Fe [46,47,48]. Only a few authors have checked for possible interference, and efforts have been limited to merely absorbance interference, rarely exploring deeply into the possible mechanism of the interaction [18]. Multiple assays should be combined for an overall cytocompatibility assessment of bioabsorbable Fe-based materials, as well as for any material.
- To investigate the mechanism of toxicity in depth, other endpoints, such as the cell cycle and gene expression profile, have to be assessed in order to define a material as biocompatible. Indeed, at the early stage, ROIs released from the material could induce oxidative stress with an increase of oxidative stress genes such as *HO-1* [37], or induce genomic DNA mutation. Carcinogenesis may be caused by depletion of antioxidant defenses, nuclear transcription factor, such as NF-kB, activation, or cell growth regulation alterations [49]. Assessing DNA alterations or damage, using a simple method such as the comet assay, can thus be fundamental for defining the potential genotoxicity of a material.
- Assessing blood compatibility is a fundamental part of defining a material as biocompatible. Blood flow across the stent surface could induce erythrocyte rupture, adsorption of plasma proteins leading to platelet activation, and finally, activation of the intrinsic coagulation pathway, resulting in thrombin activation [50]. Some studies have defined Fe-based alloys as biocompatible degradable biomaterials exclusively based on cytotoxicity results, without assessing hemolysis, platelet adhesion or coagulation [18,51]. Even if *in vitro* assays enable one to reproduce the physiological environment and the endothelium plays a key role in platelet activation, the blood compatibility test is a first step for prescreening a material. Few authors performed platelet adhesion or haemolysis assays, as indicated in Table 1. Overall, investigators showed a good *in vitro* blood compatibility for Fe-based materials, with a hemolysis rate less than 5%, according to the ISO standard ISO 10993-4, and an anti-platelet adhesion property in comparison with 316 L stainless steel.
- Finally, all authors have used healthy cell lines. As the target tissue is by definition diseased when a stent is implanted, the impact of Fe corrosion on cells from patients with coronary disease or on cells from ApoE mice, that develop atherosclerosis, might be more appropriate [52]. Messer *et al.* [53] showed, moreover, that the presence of monocytes *in vitro*, as an indicator of inflammatory disease, decreased the corrosion rate of stainless steel, demonstrating the importance of addressing the interaction of candidate implant materials with multiple components of atheromatous tissue.

In conclusion, most *in vitro* experimental protocols do not integrate the complexity of chemical forms that may arise upon corrosion, i.e., cell viability should be tested after exposure to solid material directly or indirectly through a membrane, to leachates or soluble salts of the metal. Investigators have rarely addressed mechanisms of toxicity, or the relationship with biodegradation products, and the cell culture model is not always correctly justified. Progress in this field has largely been empiric rather than supported by a detailed understanding of the interplay between physicochemical and biological phenomena involved in tissue compatibility or response.

### 3.2. In Vivo Tests

As in the human body, the implant is subjected to precise degrees of shear stress, pH, temperature and a continuous blood flow. Therefore, *in vitro* tests are useful only for prescreening or predicting the biocompatibility of a material. Indeed, most cellular tests present some limitations as they involve a static environment with an accumulation of corrosion products, whereas under *in vivo* conditions there is clearance by the blood flow [59]. Some authors attempted to reproduce the microenvironment of the stent more faithfully by using an ex vivo blood vessel bioreactor [60]. However, in this system, there is no plasma clearance of the released metal species and the degradation rate appeared too high, probably due to the absence of blood proteins. To simulate the surgical implantation, it is therefore necessary to perform animal biocompatibility tests prior to clinical implementation. *In vivo* experiments, however, are influenced by many factors such as species, sex, age, diet, activity, hormonal variations, and the need to be well consolidated before drawing conclusions [61,62].

**Figure 2:**
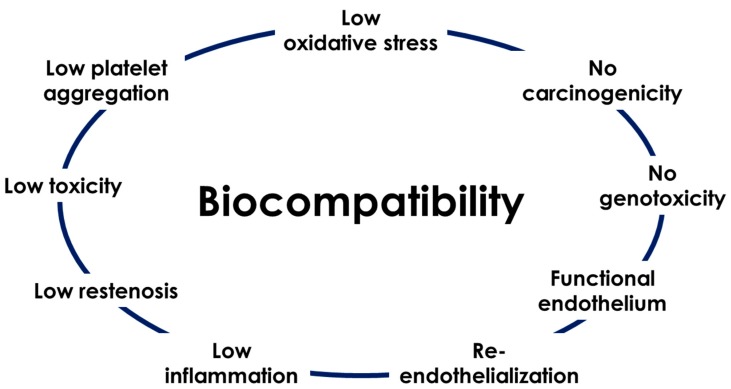
Principal parameters to define a biomaterial as biocompatible.

Biocompatibility is even more complex to define *in vivo* than *in vitro*. As illustrated in Figure 2, it is a combination of low classical adverse effects of permanent material, i.e., restenosis or thrombosis, but also low inflammation, toxicity and oxidative stress. Moreover, as other metals like arsenic, cobalt, chromium and nickel are known to be carcinogens, it is suitable to test the carcinogenicity of biodegradable materials [30]. Assessment of the genotoxic activity of the material is equally relevant in order to avoid alterations in DNA or chromosomal structure or other DNA or gene damage that results in permanent changes in cell function and ultimately carcinogenesis [63]. Finally, the reformation of a complete and functional endothelium is indispensable after the implantation. A new endothelium starts to be formed immediately after stenting (*in vivo* studies showed a neoendothelium after one week of implantation [59]), but regenerated cells have to be healthy. A functional endothelium is key to preventing neoatherosclerosis and previous studies on drug-eluting stents revealed a functionally incompetent regenerated endothelium [64]. Additionally, optimal blood compatibility can only be achieved by a monolayer of normal endothelial cells [65].

**Figure 3:**
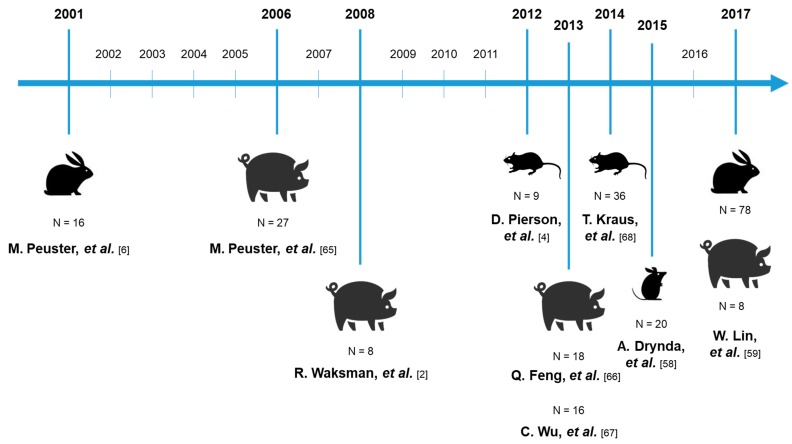
Timeline of *in vivo* Fe-based materials implantations. N represents the number of animals use.

Iron (ARMCO quality, >99.5% Fe) was the first biodegradable metal used in an animal model (Figure 3). Since 2001, investigators performed short (few days) or long-term (up to 53 months) *in vivo* studies using rabbit, minipig, rat and mouse models. Table 2 summarizes *in vivo* studies with Fe or Fe-based vascular implants (2001–2017). The first Fe stent implantation was in the descending aorta of rabbits, and after 18 months, no signs of inflammation, neointima proliferation, or systemic toxicity were recorded [6]. However, the authors documented an accumulation of degradation products (granular brownish material) at the junctions of the stent struts and a mild perifocal inflammatory reaction in one case 6 months after implantation. Five years later, the same group implanted a stent in the descending aorta of minipigs, and after histopathological analysis, they observed no signs of iron overload or organ toxicity [66]. In 2008, Waksman *et al.* [2] reported that short-term Fe stent implantation in the coronary artery of juvenile domestic pigs was associated with less neointima formation than Co–Cr stenting. Q. Feng *et al.* [67], in 2013, compared the corrosion rate of pure Fe with nitrided Fe and revealed that, while the surface area coverage of corrosion products increased from 3 to 12 months post-implantation, the inflammation score decreased. Wu *et al.* [68] in the same year performed a biocompatibility analysis one month after Fe implantation in mini-swine and Fe staining emerged positive in the spleen without signs of toxicity. In 2014, Fe-based alloys found implementations also in osteosynthesis and cylindrical pins were implanted into the rat femur until 52 weeks, showing that the tissue surrounding Fe implantation contains a significant amount of Fe ions but without toxicity [69]. Subcutaneous implantations in mice models did not allow the material to corrode, so any prediction about the suitability of Fe-based materials was not possible [58]. Finally, more recently, a large study was conducted by Lin *et al.*, which involved nitrited Fe scaffold implantation in several abdominal aortas of rabbits and minipigs. The authors reported numerous yellow-brown corrosion products in somatic cells or macrophages, and gross dissection observations showed slight inflammation in local tissues after up to 36 months implantation in rabbits, and after 53 months implantation in the porcine model [59].

The main aim of these *in vivo* works was to evaluate the degradation rate of the material and/or the biocompatibility. In all studies, the degradation rate of pure iron was found to be insufficient because large portions of the stent remained intact 1 year after the implantation, and many efforts are currently set up to increase the corrosion rate, e.g., by changing the alloy composition or the metal structure [70]. Fe *in vivo* corrosion is known to be influenced by the formation of calcium phosphate passivation layers on the surface, which reduce access to oxygen, and by the arterial environment, as shown by Pierson *et al.* [4]. These authors demonstrated that blood-contacting luminal Fe implants had a reduced corrosion rate compared to material encapsulated within the arterial wall extracellular matrix.

The attraction for Fe for biodegradable implants is partially due to the deep knowledge on its uptake, transport and excretion. It is important to known that Fe cannot be actively excreted from the body. Fe homeostasis is dependent on the regulation of its resorption from the intestinal mucosa [71]. Following intestinal resorption, Fe^3+^ binds to the transferrin. In an adult human, the total Fe binding capacity is up to 12 mg, and normally Fe^3+^ saturates 1/3 of the total transferrin. If transferrin reaches its total capacity to bind Fe, Fe ions will gradually start to bind plasma proteins, mainly albumin. Fe cannot be excreted by the renal system in this form, except if chelate-building agents are administered to force Fe out of the protein [71]. However, as mentioned above, this is unlikely to happen in view of the slow degradation rate of Fe-based materials.

Concerning the biocompatibility, all investigators defined tested materials as suitable and safe for cardiovascular applications, without signs of local or systemic toxicity, or restenosis, and able to induce a good re-endothelialization. Only Feng *et al.* in 2013 showed evidence of inflammation at 3–6 months post-implantation [66]. However, defining a material as promising requires a full assessment of various parameters. Overall, authors assessed the biocompatibility mainly by performing global classical histopathological analysis of organs such as the myocardium, brain, heart, lung, spleen, liver and kidney, demonstrating no signs of iron overload or iron-related organ toxicity [2,66,68]. However, as an implant is very light (about 40 mg) and the baseline Fe-load of blood is high (447 mg/l), the Fe released from the stent is negligible and, therefore, unlikely to cause systemic toxicity, even after implantation of multiple stents [71,72]. The potential Fe toxicity might thus mainly concern cells directly in contact, or close to the implant. It has been already demonstrated for Mg-implants that the accumulation of degradation products in the vessel wall leads to medial swelling, neointimal proliferation, and ex-stent restenosis [73,74]. Additional investigations on the interactions between corrosion products and tissue near Fe implants are required. The key parameter is to assess the mechanism of corrosion *in vivo* and show how this degradation process can induce an inflammatory response and/or toxicity both locally or systemically. Moreover, as described above, Fe corrosion implies the release of deleterious ROIs, which immediately react with surrounding tissue, making the local toxicity analysis even more relevant. So far, *in vivo* oxidative stress, endothelial dysfunction, carcinogenicity or genotoxicity have not been evaluated for biodegradable Fe-based materials. Recently, a Fe-based scaffold went, nevertheless, through a First-in-Man trial (Lifetech Scientific sponsor, Shenzhen, China) [60].

## 4. Conclusions

This review discusses the cellular and animal biocompatibility of Fe and Fe-based materials for cardiovascular applications. A critical point of view regarding the current approaches used to assess the biocompatibility of Fe-based materials is proposed.

The cellular experiments often overlooked the corrosion process, mostly focusing on eluates, and did not integrate the complexity of chemical forms that may arise upon corrosion. The *in vitro* assays rarely address possible mechanisms of toxicity, the cellular model is often not appropriate and its biological significance is assumed rather than demonstrated. Although the first Fe stent was implanted almost twenty years ago, few *in vivo* studies on Fe and Fe-based alloy implants have been published. While systemic toxicity has been acutely scrutinized, local toxicity has not been deeply investigated. Endothelial dysfunction, oxidative stress, carcinogenicity or genotoxicity have not been assessed in this context.

## Figures and Tables

**Table 1:** Overview on the main *in vitro* assays performed with Fe-based materials for stenting applications.

Metallic Materials	Form of the Material	Surface State	Cells Type	Direct/Extracts Test	Viability/Metabolic Activity Test	Blood Compatibility	References
Pure Fe	Massive samples	Mechanically polished	HUVECs	Extracts	WST-8	NA	S. Zhu, 2009 [7]
Fe; Mn; Fe35Mn	Particles	/	3T3	Insert *	WST-1	NA	H. Hermawan, 2010 [51]
As-electroformed Fe, annealed E-Fe and annealed CTT-Fe	Massive samples	Polished with SiC 1000–4000 & 0.05 µm alumina paste	Rat VSMCs	Extracts	WST-1	NA	M. Moravej, 2010 [54]
Pure Fe	Massive samples	Polished up to 1 µm SiC	Mouse bone marrow stem cells	Extracts	MTT	Platelet adhesion/haemolysis assays	E. Zhang, 2010 [55]
Bulk nanocrystalline pure Fe	Massive samples	Polished up to 2000 grit	L-929, rodent VSMC, ECV304	Extracts	MTT	Haemolysis assay	F.L. Nie, 2010 [43]
Fe alloyed by different elements (Mn, Co, Al, W, Sn, B, C & S): as cast	Massive samples	Polished up to 2000 grit	L-929, rodent VSMC, ECV304	Extracts	MTT	Platelet adhesion/haemolysis assays	B. Liu, 2011 [44]
Fe–21Mn–0.7C; Fe–21Mn–0.7C–1Pd	Massive samples	Polished with 2400 grit SiC	HUVECs	Extracts	NR; MTT	NA	M. Schinhammer, 2013 [15]
Pure Fe	Massive samples	Polished to 2000 grit	L929, ECV304	Extracts	MTT	Platelet adhesion/haemolysis assays	J. Cheng, 2013 [56]
Pure Fe	Particles	/	BALB/3T3	Insert *	WST-1	NA	A. Purnama, 2013 [57]
Pure Fe, Fe–Pd and Fe–Pt composites	Massive samples	Polished to 2000 grit	L-929, human VSMC and ECV304	Extracts	MTT	Platelet adhesion/haemolysis assays	T. Huang, 2014 [45]
Pure Fe; nitrited pure Fe	Stent; foils	Stent electrochemically polished, foils mechanically polished	L-929, human VSMC and HUVECs	Direct/Indirect/Extracts	MTT	NA	W. Lin, 2015 [35]
FeMn 0.5 wt %, FeMn 2.7 wt %, and FeMn 6.9 wt %; pure Fe	Massive samples	Polished with 2500 grit	Primary human ECs and SMCs from umbilical cord veins	Direct	Live/Dead	NA	A. Drynda, 2015 [58]
Pure Fe	Massive samples and particles	/	BALB/c 3T3	Direct/Extracts	Acridine orange dye	NA	N.S. Fagali, 2017 [36]
Pure Fe	Particles	/	HUVECs, HAoECs, HAoSMCs, HCASMCs	Direct/Extracts	WST-1; ATP	NA	E. Scarcello, 2019 [37]

CTT: casting and thermomechanical treatment; NA: not available; NR: neutral red. * particles added into 3 µm tissue culture inserts.

**Table 2:** Overview of the main *in vivo* studies with implanted Fe-based materials for stenting applications.

Material	Form of the Material	Surface State	Dimension of the Material (Diameter/Length; mm)	Animal Model	Number of Animal	Implantation Site	Duration of the Study	Application	Analysis	Results	References
Pure Fe (ARMCO quality)	Stent	Polished to achieve a strut thickness of 100–120 µm	3–6/16	New Zealand white rabbits	16	Descending aorta	6, 12, 18 months	Coronary stent	Angiography	No thromboembolic complications, no significant neointimal proliferation, no pronounced inflammatory response, and no systemic toxicity	M. Peuster, 2001 [6]
Pure Fe (ARMCO quality)	Stent	Electropolished to achieve a strut thickness of 120 µm	8/20	Minipigs	27	Descending aorta	1–360 days	Coronary stent	Histomorphometry and quantitative angiography analysis	No signs of iron overload or iron-related organ toxicity, no evidence for local toxicity	M. Peuster, 2006 [66]
Pure Fe	Stent	/	1.1:1 to 1:1.2 stent/artery diameter ratio	Juvenile domestic pigs	8	Proximal left anterior descending, left circumflex artery, or right coronary artery	28 days	Coronary stent	Histochemistry, vessel morphometry	No adverse effects in the persistent areas	R. Waksman, 2008 [2]
Pure Fe	Wire	/	0.25/20	Male Sprague Dawley rats	9	Abdominal aorta	22 days; 1.5, 3, 4.5, or 9 months	Coronary stent	Histological examination	Critical role of the arterial environment in directing the corrosion behavior of biodegradable metals	D. Pierson, 2012 [4]
Pure Fe and nitrided Fe	Stent	Electrochemically polished	8/20	Minipigs	18	Left and right iliac arteries	1, 3, 6 and 12 months	Coronary stent	Histological examination	No thrombosis or local tissue necrosis; decreased inflammation from 3-6 to 12 months post-operation	Q. Feng, 2013 [67]
Nitriding Fe	Stent	/	3/18	Minipigs	8 Fe, 8 Co-Cr	Coronary artery	28 days	Coronary stent	Coronary angiography, endothelialization and histological observation	No signs of organ toxicity	C. Wu, 2013 [68]
FeMn 0.5 wt %, FeMn 2.7 wt %, and FeMn 6.9 wt %; pure Fe	Cylindrical plate	Polished with abrasive papers 800, 1200, and 2500 grains	3/1.4 (height)	NMRI mice	20	Subcutis resting on the fascia of the gluteal muscle	3, 6, 9 months	Cardiovascular application	Histological examination	No significant corrosion was detectable, not possible to make serious predictions	A. Drynda, 2015 [58]
Fe 0.074 wt%N; pure Fe; 316L stainless steel	Scaffold	Electrochemically polished	3/18	New Zealand white rabbits	78	Abdominal aorta	7 days; 1, 4, 6, 9, 12, 24, 36 months	Coronary stent	Endothelialization and histopathologic observation	No adverse effects, homogeneous endothelial coverage, slight inflammatory response	W. Lin, 2017 [59]
Fe 0.074 wt%N	Scaffold	Electrochemically polished	3/18	Tibet minipigs	8	Left anterior descending, coronary artery and right coronary artery	33, 53 months	Coronary stent	Gross observation and histopathology analysis on the organs and tissue	No abnormalities found for the organs and no pathologic changes	W. Lin, 2017 [59]
